# A Functional *Henipavirus *Envelope Glycoprotein Pseudotyped Lentivirus Assay System

**DOI:** 10.1186/1743-422X-7-312

**Published:** 2010-11-12

**Authors:** Dimple Khetawat, Christopher C Broder

**Affiliations:** 1 Department of Microbiology and Immunology, Uniformed Services University, Bethesda, Maryland 20814, USA

## Abstract

**Background:**

Hendra virus (HeV) and Nipah virus (NiV) are newly emerged zoonotic paramyxoviruses discovered during outbreaks in Queensland, Australia in 1994 and peninsular Malaysia in 1998/9 respectively and classified within the new *Henipavirus *genus. Both viruses can infect a broad range of mammalian species causing severe and often-lethal disease in humans and animals, and repeated outbreaks continue to occur. Extensive laboratory studies on the host cell infection stage of HeV and NiV and the roles of their envelope glycoproteins have been hampered by their highly pathogenic nature and restriction to biosafety level-4 (BSL-4) containment. To circumvent this problem, we have developed a henipavirus envelope glycoprotein pseudotyped lentivirus assay system using either a luciferase gene or green fluorescent protein (GFP) gene encoding human immunodeficiency virus type-1 (HIV-1) genome in conjunction with the HeV and NiV fusion (F) and attachment (G) glycoproteins.

**Results:**

Functional retrovirus particles pseudotyped with henipavirus F and G glycoproteins displayed proper target cell tropism and entry and infection was dependent on the presence of the HeV and NiV receptors ephrinB2 or B3 on target cells. The functional specificity of the assay was confirmed by the lack of reporter-gene signals when particles bearing either only the F or only G glycoprotein were prepared and assayed. Virus entry could be specifically blocked when infection was carried out in the presence of a fusion inhibiting C-terminal heptad (HR-2) peptide, a well-characterized, cross-reactive, neutralizing human mAb specific for the henipavirus G glycoprotein, and soluble ephrinB2 and B3 receptors. In addition, the utility of the assay was also demonstrated by an examination of the influence of the cytoplasmic tail of F in its fusion activity and incorporation into pseudotyped virus particles by generating and testing a panel of truncation mutants of NiV and HeV F.

**Conclusions:**

Together, these results demonstrate that a specific henipavirus entry assay has been developed using NiV or HeV F and G glycoprotein pseudotyped reporter-gene encoding retrovirus particles. This assay can be conducted safely under BSL-2 conditions and will be a useful tool for measuring henipavirus entry and studying F and G glycoprotein function in the context of virus entry, as well as in assaying and characterizing neutralizing antibodies and virus entry inhibitors.

## Background

Hendra virus (HeV) emerged in 1994 in two separate outbreaks of severe respiratory disease in horses with subsequent transmission to humans resulting from close contact with infected horses. Nipah virus (NiV) was later determined to be the causative agent of a major outbreak of disease in pigs in 1998-99 along with cases of febrile encephalitis among people in Malaysia and Singapore who were in close contact exposure to infected pigs (reviewed in [1,2]). Phylogenetic analysis revealed that HeV and NiV are distinct members of the *Paramyxoviridae *[3,4] and are now the prototypic members of the new genus *Henipavirus *within the paramyxovirus family [4]. Pteropid fruit bats, commonly known as flying foxes in the family *Pteropodidae*, are the principal natural reservoirs for both NiV and HeV (reviewed in [2]) however recent evidence of henipavirus infection in a wider range of both frugivorous and insectivorous bats has been reported [5,6].

Since their identification, both HeV and NiV have caused repeated spillover events. There have been 14 recognized occurrences of HeV in Australia since 1994 with at least one occurrence per year since 2006, the most recent in May 2010. Every outbreak of HeV has involved horses as the initial infected host, causing lethal respiratory disease and encephalitis, along with a total of seven human cases arising from exposure to infected horses, among which four have been fatal and the most recent in 2009 (reviewed in [2]) [7-9]. By comparison there have been more than a dozen occurrences of NiV emergence since its initial recognition, most appearing in Bangladesh and India (reviewed [2]) and the most recent in March 2008 [10] and January 2010 [11]. Among these spillover events of NiV the human mortality rate has been higher (~75%) along with evidence of person-to-person transmission [12,13] and direct transmission of virus from flying foxes to humans via contaminated food [14].

In contrast to other paramyxoviruses, NiV and HeV exhibit an extremely broad host tropism and in addition to bats, horses, pigs and humans, natural and/or experimental infections have also been reported in cats, dogs, guinea pigs, hamsters (reviewed in [2]), ferrets [15] and some nonhuman primates, the squirrel monkey [16] and the African green monkey [17,18]. In those hosts susceptible to henipavirus-induced pathology, the disease is characterized as a widespread multisystemic vasculitis, with virus replication and associated pathology in highly vascularized tissues including the lung, spleen and brain [2,19]. Both the broad host and tissue tropisms exhibited by NiV and HeV can for the most part be explained by the highly conserved and broadly expressed nature of the receptors the henipaviruses employ, the ephrinB2 and B3 ligands [20-23] which are members of a large family of important signaling proteins involved in cell-cell interactions (reviewed in [24,25]).

NiV and HeV possess two envelope glycoproteins anchored within the viral membrane, a trimeric fusion (F) and a tetrameric attachment (G) glycoprotein (reviewed in [26]). The F glycoprotein is initially synthesized as a precursor F_0 _which is cleaved into the disulfide-linked F_1 _and F_2 _subunits by cathepsin L within the host cell [27]. The G glycoprotein consists of a stalk domain and globular head and G monomers form disulfide-linked dimers that associate in pairs forming tetramers [28]. The F and G oligomers associate within the membrane and G is responsible for engaging receptors, which in turn triggers F-mediated membrane fusion (reviewed in [26]). The F and G glycoproteins of NiV and HeV share ~88% and 83% amino acid identity and both NiV and HeV can elicit cross-reactive anti-envelope glycoprotein antibody responses [29]. It has also been demonstrated that F and G of NiV and HeV can efficiently complement each other in a heterotypic manner in cell-fusion assays [30]. The henipavirus F and G glycoproteins share many of the general structural features found in the envelope glycoproteins of other paramyxoviruses, and recently the structure of both receptor-bound and unbound forms of the globular head domain of NiV G have been reported [31,32].

Because of their highly pathogenic nature and lack of approved vaccines or therapeutics, HeV and NiV are classified as biological safety level-4 (BSL-4) select agents by the Centers for Disease Control and Prevention (CDC) and as priority pathogens by the National Institute of Allergy and Infectious Diseases (NIAID), having the potential to cause significant morbidity and mortality in humans and major economic and public health impacts (reviewed [1]). These restrictions have somewhat limited detailed studies on virus entry and their envelope glycoprotein functions in the context of a viral particle. To circumvent these restrictions, virus pseudotyping systems have been examined, where the envelope glycoproteins from one virus are incorporated into the progeny virions of another that lacks its own envelope glycoprotein(s), effectively changing the host range and tropism of the virus. For example, the F and G envelope glycoproteins of NiV have been successfully incorporated into recombinant vesicular stomatitis virus (VSV) lacking VSV G glycoprotein (VSV-ΔG) and encoding green fluorescent protein (GFP) [21,33]. Other widely employed viral pseudotyping systems are those based on retroviral vectors, and lentiviral vectors have emerged as promising tools for a variety gene-delivery studies and can efficiently transduce proliferating as well as quiescent cells (reviewed in [34]).

Virus pseudotyping systems have been useful for the study of otherwise highly pathogenic viral agents such as Ebola and Marburg viruses, severe acute respiratory syndrome (SARS) coronavirus (SARS-CoV) and influenza virus [35-37]. Here, building on the initial findings of Kobayashi et al., [38], who first demonstrated that simian immunodeficiency virus from African green monkey (SIVagm) could be functionally pseudotyped with the F and hemagglutinin-neuraminidase (HN) glycoproteins of Sendai virus (SeV), we demonstrate for the first time that the F and G envelope glycoproteins of NiV and HeV, a cellular protein receptor using paramyxovirus, can also be functionally pseudotyped into lentivirus particles using either a luciferase or GFP reporter gene encoding HIV-1 genome. These HIV-1 based, henipavirus glycoprotein pseudotyped particles exhibited the same cellular tropism characteristics as authentic NiV and HeV, and virus entry was specifically inhibited by antiviral agents that target the henipaviruses. The pseudotyped particles could be readily concentrated by ultracentrifugation without any loss of infectivity, and using this system we also examined the incorporation of F and G glycoproteins into virions, and explored the infectivity and pseudotyping efficiency of cytoplasmic tail truncated versions of F. This lentivirus-based henipavirus glycoprotein pseudotyped particle infection assay can also be conducted safely under BSL-2 conditions and will be a useful tool for measuring henipavirus entry and for studying F and G glycoprotein function in the context of virus particle entry, as well as in assaying and characterizing neutralizing antibodies and virus entry inhibitors.

## Results

### Henipavirus F and G envelope glycoprotein pseudotyped lentivirus particles

It is often desirable to study the functions of viral envelope glycoproteins that are involved in attachment, membrane fusion and entry in the context of a viral particle. For example, infectivity experiments using virus particles can confirm observations made from cell-cell fusion assays, studies on virus tropism, or during the characterization of antiviral agents targeting various stages in the virus entry process [39]. However, work with infectious henipaviruses is restricted to BSL-4 containment which raises both cost and safety issues. To counter this limitation, we sought to develop a henipavirus envelope glycoprotein pseudotyping system using reporter gene-encoding lentivirus vectors, which would provide a virus entry assay based on the function of the F and G glycoproteins that could be safely and routinely carried out under BSL-2 conditions.

**Figure 1:**
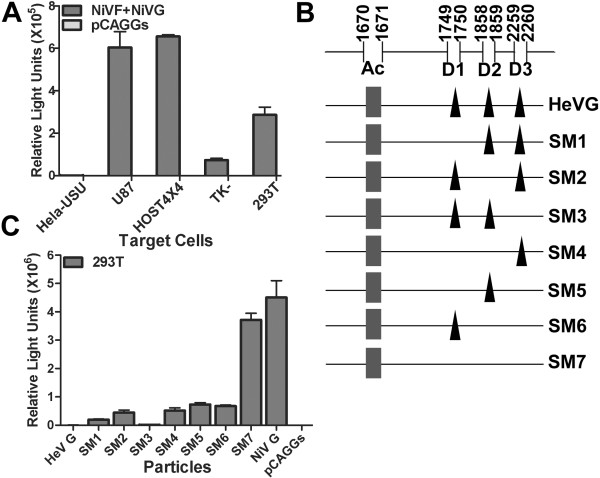
**Henipavirus F and G bearing pseudotyped lentivirus particles**. (A) Infection assay with NiV F and G glycoprotein bearing virus particles. Virus particles were prepared in 293T cells by co-transfecting the pNL4-3-Luc-E-R^+ ^HIV-1 backbone along with the NiV F and G encoding vectors, or with empty vector (pCAGGs). Culture supernatants were collected 36 hr post-transfection and filtered through a 0.45 μm filter and the pseudovirus preparations were normalized by p24 ELISA. The pseudovirus preparations were used to infect receptor positive and negative cells in triplicate wells and at 48 hr post infection, cells were lysed and assayed for luciferase reporter gene activity as described in the Methods. (B) Diagram of the panel of splice site mutants of the HeV G gene cloned into the pCAGGs vector. Putative splice donor sites are presented as black triangles and the splice acceptor site as grey squares. (C) Infection assay using pseudotyped virus particles prepared with HeV F along with (left to right) HeV G (wild-type) or each of the seven HeV G splice site mutants (SM1 - SM7); NiV G (wild-type); or empty vector (pCAGGs). Error bars indicate the standard error of the mean from triplicate wells.

To test this possibility, pseudotyped retrovirus particles were produced by transfection using pNL4-3-Luc-E-R^+^, a plasmid containing the HIV-1 proviral clone NL4-3 which encodes luciferase and does not produce the HIV-1 envelope glycoprotein [40] along with pCAGGs expression vectors encoding the NiV or HeV F and G glycoproteins. The preparations of henipavirus glycoprotein pseudotyped virus particles and control virus particles lacking the glycoproteins were normalized for p24 content by ELISA (see Methods) and used to infect several human cell lines, 293T, U87, HOSX4T4 and TK^-^, long known to be permissive for henipavirus-mediated cell-cell fusion [30,41] and the henipavirus receptor (ephrinB2 and B3) negative and fusion and infection resistant cell line HeLa-USU [20]. Pseudotyped virus particles generated with the NiV F and G glycoproteins were able to infect and produce luciferase reporter gene activity at various levels on all permissive receptor expressing cells (Figure 1A) while no signal was observed with the receptor negative HeLa-USU or with control virus particles generated by transfection with empty vector (pCAGGs). Surprisingly however, virus particles produced using the pCAGGs expression plasmids encoding the HeV F and G glycoproteins were consistently non-functional as measured by luciferase activity (data not shown). The expression vector pCAGGs is a mammalian expression vector with the cytomegalovirus (CMV) immediate early enhancer linked with the chicken β-actin promoter (CAG promoter) [42]. It has an intron with the splice acceptor site from the rabbit β-globin gene, which results in the splicing of the pre-mRNA, increasing the stability of the expressed mRNA and enhancing the production of an encoded protein. Although these features make pCAGGs an efficient vector for the expression of genes in the nucleus, we found it problematic for the expression of the HeV G glycoprotein, an RNA virus gene normally expressed in the cytoplasm of an infected cell, and expression levels of HeV G were significantly lower in comparison to NiV G in the same system (data not shown). Analysis of the HeV G gene cloned in pCAGGs using splice site prediction software from EMBL-EBI http://www.ebi.ac.uk/asd-srv/wb.cgi?method=7 revealed 3 possible splice sites within HeV G coding region (Figure 1B), while none were present in the NiV G glycoprotein pCAGGs construct (Additional file 1: **Fig. S1**). Mutations were introduced by site-directed mutagenesis to remove the predicted splice sites singly or in different combinations, keeping the amino acid coding sequence unaltered, and a panel of seven (SM1 - SM7) HeV G mutant clones were generated (Figure 1B).

The HeV G splice mutant constructs were then tested for expression by plasmid transfection which indicated that the removal of these predicted splice sites improved HeV G glycoprotein production, and removal of all three sites was optimal, and mRNA expression and alternative splicing patterns were confirmed by Northern blot analysis (results not shown). A series of pseudotyped virus particles were prepared using HeV F along with each of HeV G splice mutants (SM1 - SM7). In addition, control virus particles were also prepared using HeV F along with empty vector (pCAGGs), wild-type HeV G, or wild-type NiV G. This series of pseudotyped virus particles were then used to infect 293T target cells, and as shown in Figure 1C, the HeV G splice mutant SM7 (3 putative splice sites removed) in combination with HeV F was able to produce functional pseudotyped particles, as measured by luciferase reporter gene activity, to signal levels comparable to NiV F and G bearing particles (Figure 1A). The remainder of the HeV G splice mutants (SM1 - SM6) did show low levels of reporter gene signal, whereas the wild-type HeV G did not. These results demonstrate that the splice site removal by mutation in HeV G-SM7 restores the ability of HeV G to be expressed in the context of pCAGGs, thus allowing its incorporation into the lentivirus particles. In addition, functional particles were also generated using HeV F in heterotypic combination with NiV G, confirming the previous heterotypic cell-cell fusion activities observed with the henipaviruses [30]. The heterotypic pseudotyped particles yielded reporter gene activity essentially equivalent to the HeV G-SM7 and HeV F particles (Figure 1C) and similar to the signals obtained with NiV F and G bearing particles (Figure 1A).

**Figure 2:**
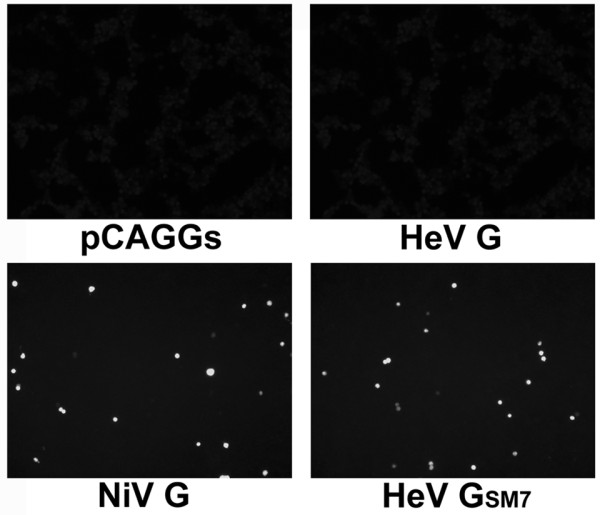
**Henipavirus F and G bearing pseudovirus infection assay with GFP-encoding lentivrus particles**. 293T cells were co-transfected with the pNL4-3-GFP-E-R^+ ^HIV-1 backbone plasmid along with either empty pCAGGs vector, homologous combinations of NiV F/G, HeV F/G, or HeV F with HeV G_SM7_. The supernatants were collected 36 hr post-transfection and processed as detailed in the methods. Receptor positive 293T cells were infected with pseudovirions and scored for transduction efficiency by counting the number of GFP positive green cells 48 hr post-infection using Olympus IX81 fluorescent microscope.

To confirm these findings and demonstrate an expanded utility of the henipavirus envelope glycoprotein pseudotyping systems, NiV and HeV F and G glycoprotein bearing lentivirus particles were prepared with the GFP reporter gene encoding construct pNL4-3-GFP-E-R^+ ^and used to infect receptor positive 293T cells (Figure 2). Here, productively infected cells were visualized using a fluorescent microscope 48 hrs post-infection and fluorescent cells were observed only in those cells infected with pseudotyped virions prepared with either NiV F and NiV G or HeV F and HeV G_SM7_. No GFP expressing cells were observed in those wells infected with virions prepared with empty vector (pCAGGs) or virus particles prepared with HeV F and wild-type HeV G.

### Specificity of henipavirus envelope glycoprotein pseudotyped lentivirus particles

**Figure 3:**
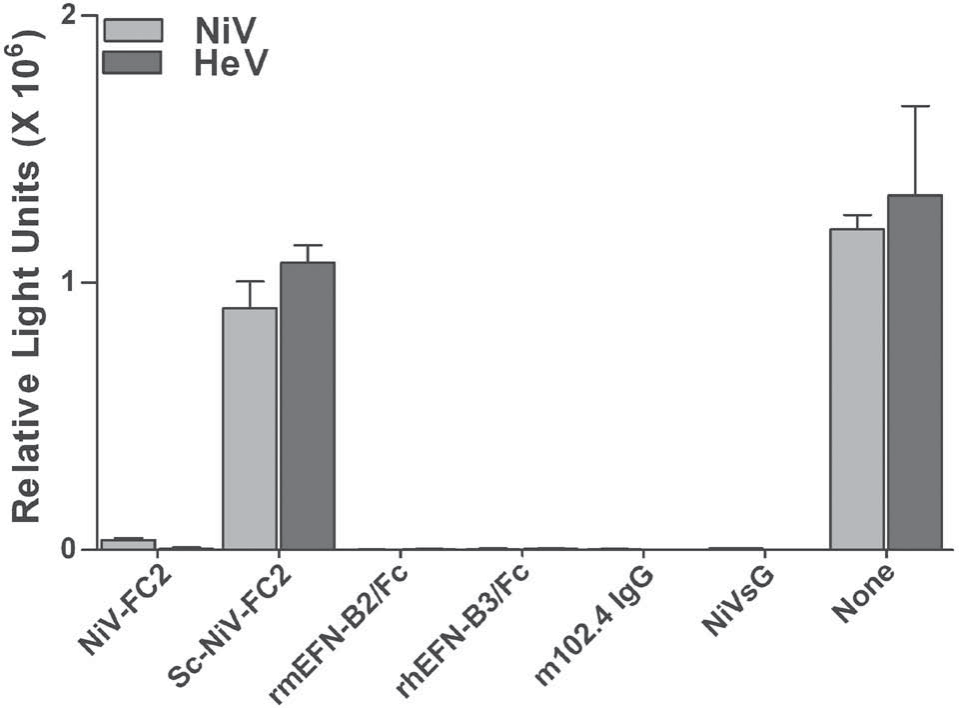
**Infection specificity of henipavirus F and G bearing pseudovirions**. The HeV and NiV envelope glycoprotein pseudotyped virus particles were preincubated with 2 μg of NiV-FC2, Sc-NiV-FC2, soluble, murine ephrin-B2, or soluble human ephrin-B2, mAb m102.4 IgG, recombinant NiV sG, or nothing (control), for 1 hr at 4°C and then receptor positive 293T cells were infected (transduced) with the various treated pseudotyped virus preparations in triplicate wells. After 1 hr incubation, complete media was added and infections were continued for an additional 48 hrs. Cells were then lysed and assayed for luciferase reporter gene activity as described in the Methods. Error bars indicate the standard error of the mean from triplicate wells.

To examine the cellular infection specificity of the HeV and NiV F and G pseudotyped particles, several henipavirus specific reagents capable of blocking virus infection were tested for their ability to inhibit the infection of the henipavirus pseudotypes. Virus particles were prepared as before and then mixed with various inhibitors (Figure 3). The henipavirus specific peptide fusion inhibitor NiV-FC2, a 36 amino acid peptide corresponding to the henipavirus heptad repeat region 2 (HR-2) of the F glycoprotein [39,41], completely blocked the entry of the henipavirus pseudotypes as measured by luciferase reporter gene activity. The NiV-FC2 peptide functions in an analogous manner to the HIV-1 specific fusion inhibitor enfuvirtide (Fuzeon™, formerly T-20) [43,44], and specifically blocks the formation of the class 1 fusion glycoprotein structure known as the 6-helix bundle of the F glycoprotein preventing F-mediated membrane fusion and subsequent virion entry. A scrambled version of the peptide (Sc NiV-FC2) was used as a negative control. Infection specificity was also examined by inhibition with the cross-reactive anti-henipavirus G glycoprotein human monoclonal antibody (mAb) m102.4 [45,46]. The m102.4 mAb neutralizes henipaviruses by specifically binding and blocking the ephrin-B2 and -B3 receptor-binding region on the henipavirus G glycoprotein. As shown in Figure 3, infection of either the HeV or NiV pseudotypes was completely blocked by mAb m102.4 confirming that their entry and resultant luciferase signal is specifically mediated by the attachment and subsequent fusion triggering functions of their henipavirus G glycoproteins. In addition, the binding and infection of the henipavirus pseudotypes to target cells could be blocked by recombinant, soluble ephrin-B2 and -B3 receptors (Figure 3). HeV and NiV F and G bearing particles pre-incubated with soluble ephrin-B2 or -B3 were unable to infect host cells as was previously shown with infectious virus [20]. Also, in a reciprocal manner, recombinant soluble NiV G (sG) could block entry of either henipavirus pseudotype as was similar to earlier observations made with HeV sG in infectious virus entry. Together, these results demonstrate the specificity of the henipavirus F and G glycoprotein bearing pseudotyped virus entry assay and its potential utility in screening specific henipavirus entry inhibitors.

### Influence of the henipavirus F glycoprotein cytoplasmic tail on processing and function

**Figure 4:**
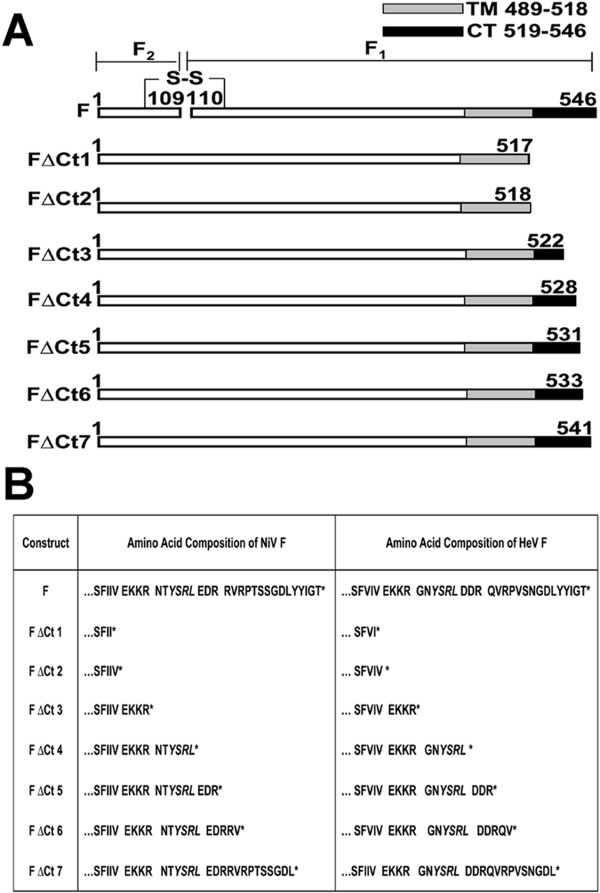
**Schematic diagram of truncation mutants in the fusion glycoprotein**. (A) A schematic representation of the F glycoprotein truncation mutants. The transmembrane and the cytoplasmic tail regions are marked along with the disulfide bond linking F_1 _and F_2_. The nomenclature for the constructs is shown on the left and the position of stop codon on the right. (B) The amino acid composition of truncation mutants near the truncation site within the F glycoprotein of both HeV and NiV.

Previous studies have demonstrated that efficient incorporation of heterologous envelope glycoproteins into HIV-1 or murine leukemia virus (MLV) particles often depended on the removal of part or all of the cytoplasmic tail domains from the pseudotyping glycoproteins [38,47-49]. To explore whether a similar feature was occurring in the henipavirus pseudotyping system here, a series of seven cytoplasmic tail truncation mutations in each henipavirus F glycoprotein were generated, designated FΔCt1 to FΔCt7, by introducing translational stop codons into the coding sequence of the NiV and HeV F gene (Figure 4). The FΔCt1 and FΔCt2 constructs of both the NiV and HeV F, differ by only one additional deleted valine residue to better ensure complete removal of the cytoplasmic tail domain.

**Figure 5:**
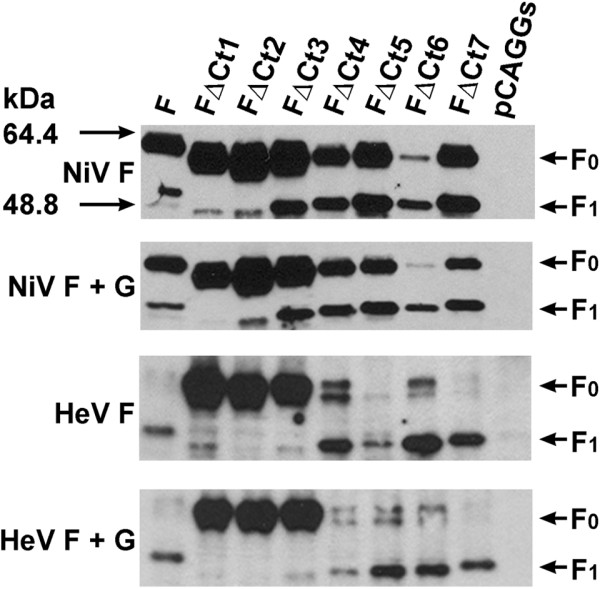
**Cell surface expression of truncation mutants of the henipavirus F glycoprotein**. The various F cytoplasmic tail truncation mutants alone or together with their G glycoprotein partner were transfected into HeLa-USU cells. At 24 hr post transfection, cell surface proteins were biotinylated and precipitated with Avidin agarose beads, and the precipitated proteins were processed for Western blot analysis as detailed in the Methods and probed using the anti F_1 _specific antisera. This experiment was performed twice and representative experiment is shown in the figure.

Because cytoplasmic tail truncations of membrane anchored proteins could affect proper folding and transport, we first examined the levels of cell surface expressed F and compared the series of truncated mutant F constructs to each wild-type F, using a cell surface biotinylation assay [50]. The series of NiV and HeV F glycoprotein mutants and each wild-type F were expressed by plasmid transfection in HeLa-USU cells, both in the presence and absence of their homologous G glycoprotein partner, and surface proteins were biotin labeled, precipitated with Avidin-agarose, and analyzed by Western blot assay using an F_1 _specific antisera (Figure 5). The wild-type NiV F_0 _precursor was cleaved and detected. FΔCt1, FΔCt2 appeared less efficiently cleaved (levels of F_1 _versus F_0_) as compared to wild-type NiV F. A significant amount of each of the NiV FΔCt3, FΔCt4, FΔCt5, FΔCt6 and FΔCt7 constructs were cleaved, with the FΔCt6 appearing highly processed although its overall expression was lower in comparison to others. The ratio of cleaved to uncleaved F (F_1 _to F_0_) on the cell surface was approximately equal (1:1) when the complete retention and endocytosis motif (YSRL) [51,52] was retained, beginning with the FΔCt4 constructs. Notably, the coexpression of NiV G did not appear to significantly alter the expression and cleavage patterns of NiV F (Figure 5).

The retention of amino acid residues from the endocytosis motif YSRL to residues EDRRV in the cytoplasmic tail appeared to allow for more efficient F_0 _processing, as evidenced by the greater levels of F_1 _observed with these NiV constructs (NiV FΔCt4 to FΔCt7) (Figure 4) in comparison to NiV FΔCt1, FΔCt2 and FΔCt3 which lack the YSRL motif. In addition, the cell surface levels of F (primarily F_0_) observed with the FΔCt1, FΔCt2 and FΔCt3 constructs appeared greater in comparison to the FΔCt4, FΔCt5, FΔCt6 and FΔCt7 constructs, and this mostly likely reflects the reduced ability of the F_0 _precursor to be endocytosed and processed by Cathepsin L [27,53]. Similar results were obtained when the series of HeV F cytoplasmic tail truncation mutants were examined in parallel, and the HeV F constructs FΔCt1, FΔCt2 and FΔCt3 revealed greater cell surface expression levels of F_0 _with less efficient processing as measured by the detection of F_1_, whereas the HeV F constructs, FΔCt4 through FΔCt7 revealed greater F_0 _precursor processing but perhaps an overall lower level of expression (Figure 5). A variable and doublet appearance of HeV F_0 _has been observed previously [30,54,55]. As with the NiV F truncation mutants the coexpression of the HeV F panel along with their HeV G glycoprotein partner did not significantly alter the HeV F expression and cleavage patterns observed in cell surface biotinylation assays.

**Figure 6:**
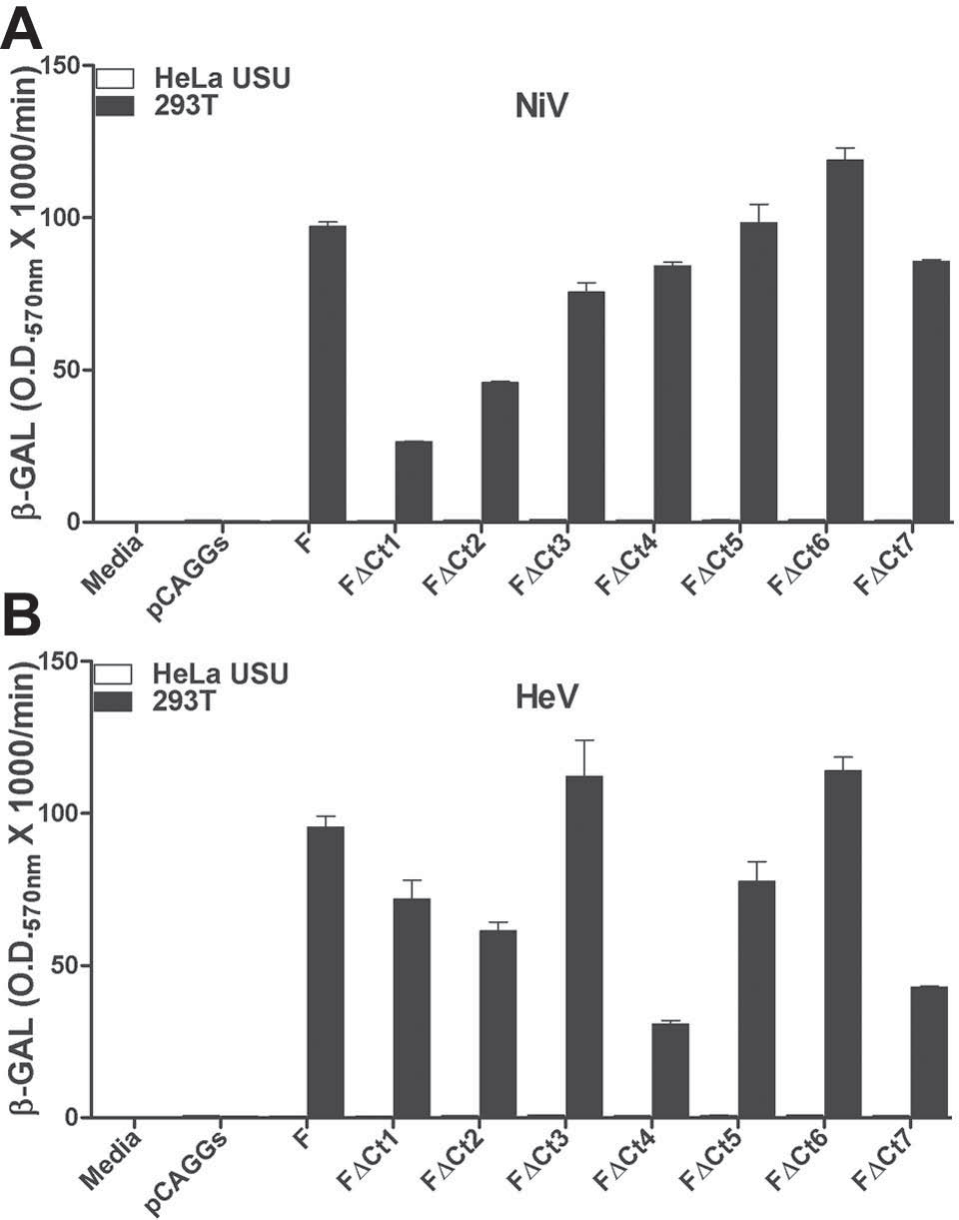
**Membrane fusion activity of the truncation mutants of the F glycoprotein**. The panels of F glycoprotein truncation mutants were assayed for their ability to mediate cell-cell fusion when co-expressed with their partner G glycoprotein in a quantitative vaccinia virus-based cell-cell fusion assays. Each F glycoprotein mutant was tested in triplicate wells in three independent experiments. Shown are the results of a representative cell-fusion assay with the F truncation mutants FΔCt1 through FΔCt7 along with wild-type NiV or HeV F as positive controls and vector only (pCAGGs) or media only as negative controls. (A) NiV G along with the panel of truncation mutants of NiV F. (B) HeV G along with various truncation mutants of HeV F. Error bars indicate the standard error of the mean from triplicate wells.

Having characterized the expression and processing of the cytoplasmic tail truncation mutants of both NiV and HeV F glycoprotein, we next examined their biological function in cell-cell membrane fusion assays. Membrane fusion was assessed using the well-characterized vaccinia virus-based, reporter-gene, cell-cell fusion assay [56]. This assay has also been used extensively in earlier reports on the characterization of HeV and NiV-mediated membrane fusion and tropism [30,41,57]. The series of F glycoprotein truncation mutants for both HeV and NiV were expressed, along with their respective partner G glycoprotein, in HeLa-USU cells (effector cells) and cell-cell fusion reactions were carried out using target cells of either receptor negative HeLa-USU (control) or fusion permissive 293T cells, and results are shown in Figure 6. For NiV F, the removal of most of the cytoplasmic tail domain from F (FΔCt1 and FΔCt2), which also reduced F_0 _processing, impaired their fusogenic potential as would be expected, whereas the fusogenic activity of NiV FΔCt3, FΔCt4, FΔCt5, FΔCt6 and FΔCt7 were either equivalent or slightly elevated in comparison to wild-type NiV F. The cell-cell fusion assay with the series of HeV F truncation mutants generated slightly more variable results in contrast to NiV F, though all possessed some fusogenic activity. In general there was only a slight reduction in fusion with HeV F, FΔCt1 and FΔCt2, while FΔCt3, FΔCt5 and FΔCt6 were essentially equivalent to wild-type HeV F, while lower fusion signals were seen with HeV FΔCt4 and FΔCt7, which could be related to an overall lower expression level as seen in Figure 5. A comparison of the results in Figure 5 and Figure 6 suggests that NiV F processing appears to correlate with cell-cell fusion signals; whereas cell-cell fusion activity was readily apparent in several HeV F truncation mutants possessing a markedly lower level of F_0 _processing, however these are independent experiments and a direct comparison may be miss-leading.

### Incorporation and function of truncated F glycoproteins into lentivirus particles

**Figure 7:**
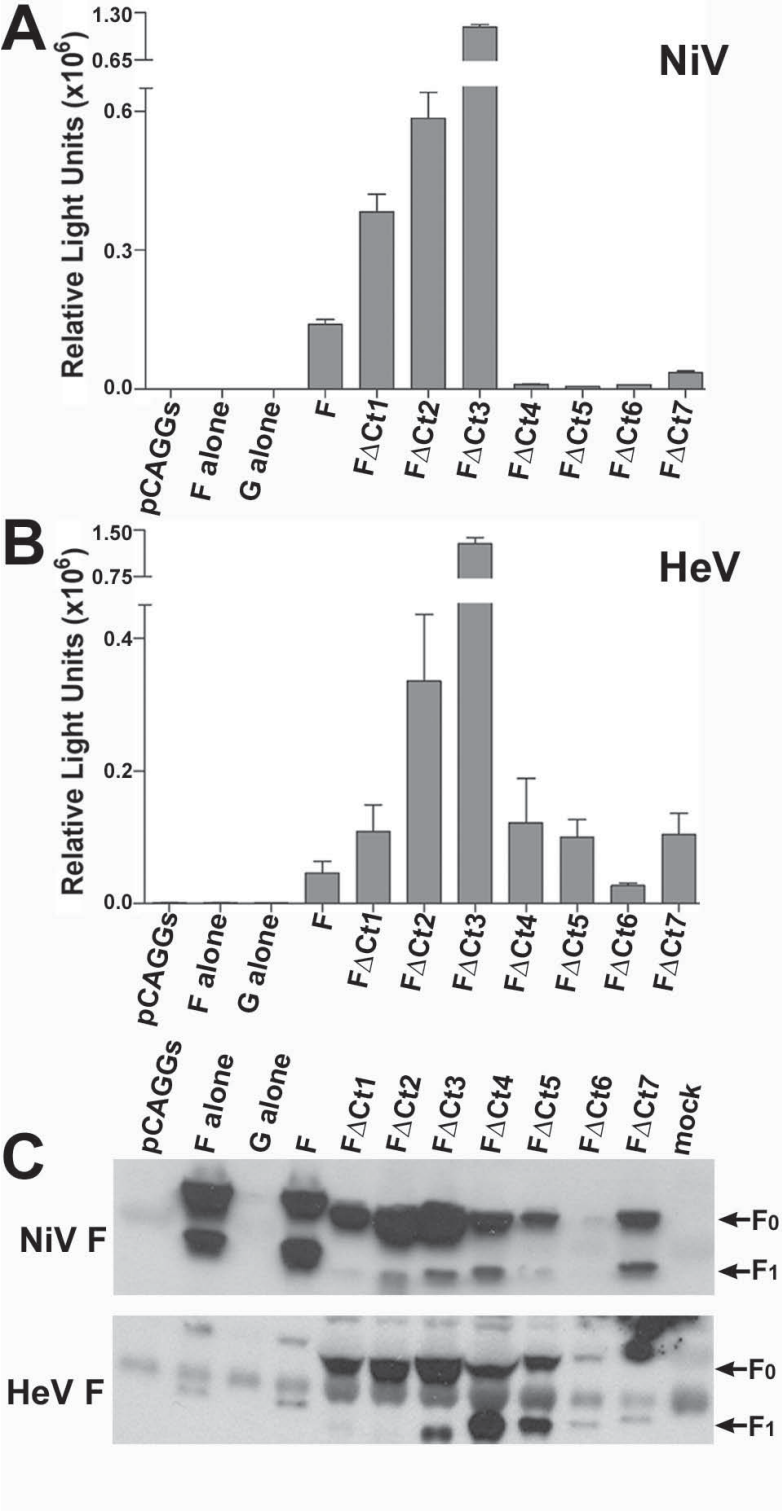
**Envelope glycoprotein incorporation efficiency and infectivity of henipavirus F and G bearing lentivirus particles**. The panel of expression plasmids encoding the NiV and HeV F glycoprotein cytoplasmic tail truncation mutants and/or their G glycoprotein partner together with the HIV-1 backbone pNL4-3-Luc-E-R^+ ^were transfected into 293T cells. The pseudovirus containing cell culture supernatants were collected 36 hr post-transfection, filtered with a 0.45 μm filter and purified through a 25% wt/vol sucrose cushion. The preparations of pseudovirions were normalized by assaying p24 content and then used to infect permissive 293T target cells. (A) Infection assay with the various NiV F cytoplasmic tail deletion mutants. (B) Infection assay with the various HeV F cytoplasmic tail deletion mutants. Error bars indicate the standard error of the mean from triplicate wells. (C) Incorporation of the various NiV and HeV F glycoproteins into the lentivirus-based pseudovirions. Equal amounts of particles, based on p24 content, were lysed and subjected to SDS-PAGE and Western blot analysis to assess the levels of incorporation of the F glycoproteins. Mock is processed supernatant prepared from cells not producing pseudovirions. This experiment was performed twice and representative experiment is shown in the figure.

We next examined the efficiency of the various cytoplasmic tail truncation mutants of the NiV and HeV F glycoproteins to be incorporated into lentivirus-based pseudotypes. Pseudotyped lentivirus particles were prepared as before using the series of cytoplasmic tail truncation mutants along with their partner G glycoprotein. Three types of control virus particles were also prepared using either empty vector (pCAGGs) or each species of wild-type F glycoprotein alone or each species of G glycoprotein alone. Pseudotyped virus particle preparations were filtered, purified by centrifugation through a sucrose cushion, normalized for p24 content by ELISA and used to infect 293T target cells. Following infection and incubation for 48 h, cells were processed and luciferase activity was measured. As shown in Figure 7A and 7B, pseudovirus particles prepared using the truncation mutants FΔCt1, FΔCt2, and FΔCt3 F glycoproteins produced significantly greater levels of luciferase activity as compared to virus particles made with wild-type F. To evaluate whether the differences in infectivity, as measured by luciferase reporter gene activity, by the various pseudovirus types correlated to the extent of incorporation of the mutant F glycoproteins into lentivirus particles, equal amounts of virus particles based on p24 content were lysed and analyzed by Western blot. This analysis revealed that incorporation of F into either the NiV or HeV pseudotyped virions was greater with the FΔCt1, FΔCt2 and FΔCt3 constructs, and that F_0 _was the predominant species present in the virions (Figure 7C). These results together with cell surface expression pattern of the NiV and HeV wild-type and truncation mutants demonstrate that, in general, the amount of incorporation of the F glycoproteins in the pseudotyped particles appears to correlate well with the level of expression of these proteins on the surface of the producer cells. This was also true in the amount of wild-type NiV or HeV F alone bearing particles which can be noted when comparing Figure 5 and Figure 7C. Removal of the endocytosis motif from the fusion protein prevents its transportation to the endosome and subsequent cleavage of F_0 _into F_1 _and F_2 _by Cathepsin L, which explains the predominance of F_0 _in the FΔCt1, FΔCt2 and FΔCt3 constructs which lack the endocytosis motif YSRL. Interestingly, in both the NiV and HeV F glycoprotein mutant series, the higher infectivity of the FΔCt1, FΔCt2 and FΔCt3 bearing pseudotypes in comparison to wild-type was notable, and might be attributed to the greater levels of incorporation of these F glycoproteins into the particles, except in the case of wild-type NiV F and G bearing particles and the reason for this later observation is unclear at present. Alternatively however, and also of interest is that the high infectivity signal and predominance of F_0 _in the pseudotypes prepared with FΔCt1, FΔCt2 and FΔCt3 could argue for a role of endocytosis followed by Cathepsin L processing of F_0 _and subsequent productive fusion and infection.

## Discussion

In the present study we have detailed a new and readily adaptable, reporter-gene containing, lentivirus-based pseudotyping system which utilizes functional F and G envelope glycoproteins of the henipaviruses; NiV and HeV. Importantly, like other virus envelope glycoprotein pseudotyping systems, this assay can be conducted safely under BSL-2, a condition which is relevant considering the otherwise highly pathogenic nature of infectious NiV and HeV. We also demonstrate, by several measures, that this henipavirus pseudotyping system faithfully recapitulated the natural NiV or HeV cell attachment and viral glycoprotein-mediated membrane fusion stages of infection.

The henipaviruses bind and infect their host cells by a specific attachment step to the cell surface expressed proteins ephrin-B2 and -B3 [20-23]. The current and widely accepted model of paramyxovirus mediated membrane fusion postulates that upon receptor binding the viral attachment glycoprotein triggers conformational changes in the F glycoprotein, a class I viral fusion glycoprotein. The receptor-induced triggering event is presumed to involve direct contacts between an attachment and fusion glycoprotein and this activation process facilitates a series of conformational changes in F and the glycoprotein transitions into its post-fusion, six-helix-bundle conformation concomitant with the merging of the viral membrane envelope and the host cell plasma membrane [26,58]. However, all of the details of the entire receptor binding and fusion activation process have yet to be defined. An important feature of many class I fusion glycoproteins is the two α-helical regions referred to as heptad repeat (HR) domains that are involved in the formation of the six-helix-bundle structure [59,60]. HR-1 is located proximal to the amino (N)-terminal fusion peptide and HR-2 precedes the transmembrane domain near the carboxyl (C)-terminus. Peptide sequences from either HR domain of the F glycoprotein of several paramyxoviruses, including HeV and NiV, have been shown to be inhibitors of the F-mediated membrane fusion step in both cell-cell fusion and virus infection assays [30,39,41,57,61-66]. Here, as has been shown with infectious virus or cell-cell fusion assays, the infection by NiV and HeV F and G lentivirus pseudotypes was completely blocked by the HR-2 based fusion inhibiting peptide (NiV-FC2) [39].

A number of other tests were also conducted to demonstrate the specificity of the henipavirus pseudotyping system in addition to using the henipavirus peptide fusion inhibitors. In competition assays, the infection of the pseudotypes could also be specifically blocked using recombinant, soluble ephrin-B2 or ephrin-B3 receptor proteins as was previously shown with both henipavirus-mediated membrane fusion as well as live virus infection assays[20]. In a similar fashion, recombinant, soluble henipavirus G glycoprotein (sG) was also able to completely inhibit the infection of either HeV or NiV pseudotypes by blocking receptor binding, which had been demonstrated previously in both henipavirus-mediated membrane fusion and live virus infection assays [28]. Finally, the infection by the NiV and HeV pseudotypes could also be completely blocked using a well-characterized, cross-reactive human mAb (m120.4) that is specific for the henipavirus G glycoprotein [15,46]. Thus, by a wide variety of well-known and well-characterized approaches the functional henipavirus envelope glycoprotein pseudotyped lentivirus assay system developed here, accurately recapitulates the receptor binding, membrane fusion and infection stages of live HeV and NiV.

Because of both the highly pathogenic features of NiV and HeV, which restricts the use of infectious virus to BSL-4 containment, and the labor intensive nature and challenges associated with a reverse genetics approach, extensive and detailed structural and functional studies on the henipavirus envelope glycoproteins in the context of a viral particle has been limited. To demonstrate the utility of the henipavirus pseudotyping system here, we generated and tested an extensive panel of cytoplasmic tail domain truncation mutants of the NiV and HeV F glycoprotein, and examined the influence of this domain of F on its ability to be incorporated into this budding particles as well as its fusion activity in the context of a viral particle.

Here, it was observed that the deletion of essentially the entire F cytoplasmic tail domain, most notably with the NiV F glycoprotein and to a lesser degree with that of HeV F, impaired their fusogenic activity in the context of a cell-cell fusion assay. These findings were in contrast with previous observations made on the envelope glycoproteins of certain lentiviruses. Studies with human immunodeficiency virus type 2 (HIV-2) and simian immunodeficiency virus (SIV) envelope (Env) glycoproteins have shown that cytoplasmic domain truncation mutants exhibit significantly enhanced Env fusogenic activity as measured by syncytium formation [67,68]. In addition, studies with murine leukemia virus have demonstrated that naturally occurring late cleavage of a small carboxy terminal sequence, designated as the R peptide or p2E, in the cytoplasmic tail results in considerably enhanced cell-to-cell fusion activity [69,70]. Whereas for a paramyxovirus F glycoprotein, cytoplasmic tail deletions in simian virus 5 (SV5) [71], Newcastle disease virus [72], and human parainfluenza virus (HPIV) type 3 (HPIV-3) revealed significantly reduced syncytium formation, except in one example with HPIV-2, where similar deletions did not affect membrane fusion [73]. Overall, with the exception of the results with HPIV-2, these studies also demonstrated that subsequent additions of parts of the deleted cytoplasmic tail sequences restored the fusogenic potential of those F glycoproteins. In the case of henipaviruses, one explanation to account for the reduced fusion activity of the entire cytoplasmic tail deleted constructs is poor endocytosis and subsequent Cathepsin L processing of F_0 _and the analysis of the surface expressed levels of NiV F_0 _versus F_1 _in the cytoplasmic tail domain truncation mutants support this conclusion, but to a lesser extent with that of the HeV F truncation mutants.

However, although the cell-cell fusogenic results with the truncation constructs of the henipavirus F glycoproteins reported here were similar to the majority of the observations made with other paramyxoviruses, whether as a result of F_0 _precursor processing or by some other mechanism, the cytoplasmic tail deleted HeV and NiV F glycoproteins in the context of the virus particle pseudotyping system, revealed an opposing result. In general, the higher levels of pseudotyped particle infectivity signal correlated with an overall greater level of incorporated F glycoprotein. Interestingly however, the highest luciferase signals in the virus infection assays also correlated with a greater level of unprocessed F_0 _in the particles, particularly with FΔCt1, FΔCt2 and FΔCt3 in which most of the cytoplasmic tail was deleted. Potentially, the greater luciferase signals in these instances (FΔCt1, FΔCt2 and FΔCt3) could be due to particle endocytosis following receptor binding [74] and subsequent F_0 _processing by Cathepsin L [27]. The pseudotyping system described here offers one system, albeit artificial, to explore the possibility of a productive early endocytic route of henipavirus infection. Taken together, this henipavirus pseudotyping system shown here offers a useful tool for measuring not only henipavirus entry and assaying and characterizing virus neutralizing antibodies and virus entry inhibitors, but also offers a highly versatile platform for studying F and G glycoprotein function in the context of a virus particle during infection, and one that can readily assay numerous variations or mutants of either or both the F and G henipavirus glycoproteins.

## Conclusions

Functional *henipavirus *envelope glycoprotein pseudotyped, reporter gene encoding, lentivirus particles could be readily produced, concentrated by ultracentrifugation and stored frozen without loss of infectivity. These *henipavirus *pseudotyped particles maintained the same cellular tropism characteristics as authentic NiV and HeV, and infection of host cells by these particles could be specifically inhibited by various antiviral agents that target the henipaviruses. This henipavirus glycoprotein pseudotyped virus infection assay can be conducted safely under BSL-2 conditions and its utility in analyzing the viral glycoprotein function, of otherwise BSL-4 restricted agents, in the context of a virus particle was demonstrated in the characterization of cytoplasmic tail truncated versions of the F glycoprotein. This new henipavirus pseudotyping system will be a useful tool for measuring HeV and NiV entry and studying their F and G glycoprotein function in the context of virus particle, as well as in assaying and characterizing neutralizing antibodies and virus entry inhibitors.

## Methods

### Cells and culture conditions

U87 and HuTK^-^143B were obtained from the American Type Culture Collection (ATCC). Recombinant human osteosarcoma cells bearing CD4 and CXCR4 (HOST4X4) were obtained from the NIH AIDS Research and Reference Reagent Program [75]. The 293T cells were obtained from Dr. G. Quinnan (Uniformed Services University). HeLa-USU cell line has been described previously [20]. HeLa-USU, U87, HOST4X4 and 293T cells were maintained in Dulbecco's modified Eagle's medium (Quality Biologicals, Gaithersburg, MD) supplemented with 10% cosmic calf serum (CCS) (HyClone, Logan, UT) and 2 mM L-glutamine (DMEM-10). All cell cultures were maintained at 37°C in a humidified 5% CO_2 _atmosphere.

### Plasmids

The HeV and NiV F and G envelope glycoproteins were transiently expressed using the mammalian expression vector pCAGGs which contains the CAG promoter and is composed of the cytomegalovirus immediate early enhancer and the chicken *β*-actin promoter [42]. The HIV-1 pNL4-3-Luc-E-R^+ ^or pNL4-3-GFP-E-R^+ ^backbone plasmids encoding the luciferase (Luc) [40] or green fluorescence protein (GFP) reporter gene were provided by Dr. R. Doms (University of Pennsylvania).

### Antibodies, recombinant proteins and peptides

The henipavirus G and F glycoproteins were detected with a cross-reactive polyclonal mouse antiserum raised against recombinant, soluble HeV G [23,50] or a rabbit polyclonal henipavirus F_1_-specific antiserum provided by Dr. L-F. Wang (Australian Animal Health Laboratory, Geelong, Australia) respectively. The human monoclonal antibody (mAb) m102.4 IgG used for inhibition of virus entry [15,45,46] was provided by Dr. D. Dimitrov (National Cancer Institute-Frederick, National Institutes of Health). The fusion inhibiting peptide NiV-FC2 corresponding to the HR2 region of NiV F and the non-fusion inhibiting scrambled control peptide Sc-NiV-FC2 have been previously described [39]. Recombinant, soluble ephrin-B2 and -B3 were from R&D Systems, Minneapolis, MN. Recombinant, soluble NiV G (NiV sG) has been previously described [76]

### Fusion (F) glycoprotein constructs and mutagenesis

Full-length cDNA clones of the NiV and HeV F glycoprotein genes [30,41] each including the Kozak consensus sequence (CCACC) appended upstream of the initial ATG [77] were subcloned into pCAGGs, generating the NiV F-pCAGGs and HeV F-pCAGGs expression vectors. The cytoplasmic tail domain truncation mutants of NiV and HeV F were generated by introducing stop codons corresponding to amino acid positions 517, 518, 522, 528, 531, 533 and 541 of the full-length F glycoprotein by standard PCR techniques. The NiV F-pCAGGs and HeV F-pCAGGs plasmids were used as templates, and the 5' primer included an external *EcoRI *site, the Kozak consensus sequence (CCACC) and F-specific sequence. The various 3' primers included F specific sequence, a stop codon at the desired location and an external *KpnI *site. All PCR products were gel purified and cloned into the TOPO vector (Invitrogen) and subsequently subcloned into pCAGGs, generating constructs NiV FΔCt1 through FΔCt7 and HeV FΔCt1 through FΔCt7 (Figure 4A**and **4B). All constructs were sequenced confirmed.

### Attachment (G) glycoprotein constructs and mutagenesis

Full-length cDNA clones of the NiV and HeV G glycoprotein genes [30,41] each including the Kozak consensus sequence (CCACC) appended upstream of the initial ATG [77] were subcloned into pCAGGs, generating the NiV G-pCAGGs and HeV G-pCAGGs expression vectors. The splice site mutations (Figure 1C) of the HeV G gene were generated by site-directed mutagenesis using the QuickChange II Site-directed Mutagenesis Kit and QuickChange Multi Site-directed Mutagenesis Kit (Stratagene, Cedar Creek, TX). The template for the mutagenesis reactions was a HeV G clone in the TOPO plasmid (Invitrogen Corp., Carlsbad, CA). For the first donor splice site D1 at nucleotide position 1749, the TTG codon for leucine was changed to CTT. The other redundant codons for leucine are CTG, CTA, CTC, and TTA, but these were not effective in removing the predicted splice site. For the second donor splice site D2 at nucleotide position 1858, the AGT codon for serine was changed to TCG. For the third donor splice site D3 at nucleotide position 2259, the GGG codon for glycine was changed to GGT. All PCR products were gel purified and cloned into TOPO and subsequently subcloned into pCAGGs. All mutation-containing constructs were sequence verified.

### Cell surface biotinylation

HeLa-USU cells grown in T25cm^2 ^flasks were transfected with the pCAGGs expression constructs of NiV and HeV F alone or along with their partner G glycoprotein constructs with Fugene reagent (Roche Diagnostics Corp, IN) for 36 hrs. Following expression, cells were rinsed three times with ice-cold phosphate buffered saline (PBS) and cell surface proteins were biotinylated using 0.25 mg/ml EZ-Link NHS-Biotin (Pierce, Rockford, IL) in PBS for 30 min at 4°C [50]. The reaction was quenched by washing the cell monolayer three times with ice-cold PBS before harvesting cells and preparation of cell lysates. Cells were lysed in 100 mM Tris-HCl (pH 8.0), 100 mM NaCl, 1% Triton X-100 and protease inhibitor at 4°C for 30 min and one-half of each lysate was incubated with 100 μl of 20% vol/vol solution of Agarose-Avidin D beads (Vector Laboratories, Inc., Burlingame, CA) in IP buffer (0.14 M NaCl, 0.1 M Tris, and 0.1% Triton) at 4°C and rotated overnight. Beads were washed twice with lysis buffer followed by one wash with DOC buffer (100 mM Tris-HCl (pH 8.0), 100 mM NaCl, 0.1% sodium deoxycholate, and 0.1% SDS). Samples were boiled in SDS-PAGE sample buffer with 2-mercaptoethanol, separated on a 4-20% Tris-Glycine gradient gel (Invitrogen), transferred to nitrocellulose, and probed with a cross-reactive polyclonal mouse antiserum to HeV G at a concentration of 1:25,000 or a rabbit polyclonal F_1 _specific antiserum at a concentration of 1:25,000.

### Cell fusion Assays

Henipavirus F and G mediated fusion activities were measured using a previously described quantitative viral glycoprotein-mediated cell-cell fusion assay [30,41,57]. Briefly, one cell population (effector cells) is infected with a recombinant vaccinia virus expressing the T7 polymerase (vTF7.3) and the other cell population (target cells) is infected with a vaccinia virus encoding the *E. coli lacZ *gene (β-Gal) gene under control of the T7 promoter (vCB21R). Cell-cell fusion between effector and target cell results in β-Gal synthesis which can be measured by specific synthetic substrate cleavage. Plasmids encoding NiV or HeV G along with their respective wild-type F glycoprotein partner or the various truncation mutants of F were transfected into HeLa-USU cells and allowed to express overnight (effector cell populations). Effector cell populations were also prepared using empty vector, pCAGGs, or NiV or HeV F glycoprotein alone as additional negative controls. The various effector cell populations were infected with vTF7.3 and a fusion permissive 293T target cell population was prepared by infection with vCB21R. Vaccinia virus infections were carried out with a multiplicity of infection of 10, suspended in media and incubated at 31°C overnight as previously described[30,41,57]. Cell fusion reactions were conducted with the various cell mixtures in 96-well plates at 37°C with a ratio of envelope glycoprotein-expressing cells to target cells of 1:1 using 2 × 10^5 ^total cells per well in a total volume of 0.2 ml per well for 2.5 h. For quantitative analyses, Nonidet P-40 was added (0.5% vol/vol) and aliquots of the cell-cell fusion lysates were assayed for β-Gal at ambient temperature with the substrate chlorophenol red-d-galactopyranoside (Roche Diagnostics Corp, IN, USA). Assays were performed in triplicate, and fusion results were calculated and expressed as rates of β-Gal activity (change in optical density at 570 nm per minute × 1,000) in an MRX microplate reader (Dynatech Laboratories, Chantilly, VA).

### Preparation of henipavirus envelope glycoprotein pseudotyped lentivirus particles

Pseudotyped, HIV-1 reporter gene encoding virus stocks were prepared by transfecting 293T cells with the reporter gene-encoding backbone plasmids pNL4-3-Luc-E-R^+ ^or pNL4-3-GFP-E-R^+ ^along with the henipavirus envelope glycoprotein encoding pCAGGs vectors. 293T cells (0.75 × 10^6^) were seeded in a 6-well flat-bottom collagen I-coated microplate (BD Biosciences, Durham, NC) and transfected with the expression plasmids using the Fugene reagent (Roche Diagnostics Corp, IN). The DNAs of the pNL4-3-Luc-E-R^+ ^or pNL4-3-GFP-E-R^+ ^along with the HeV or NiV F and G encoding pCAGGs plasmids were mixed in the ratio of 1:0.2:0.8, respectively, and added to 500 μl of serum free DMEM and 9 μl Fugene. The mixture was incubated at room temperature for 30 min and then applied to the culture of 293T cells. After 3 to 5 hr incubation at 37°C, transfected cells were washed extensively with DMEM and incubated for additional 24-48 hr with 1 ml of DMEM-10, at 37°C in 5% CO_2_. The supernatants from virus particle producing cultures were then collected and were clarified by centrifugation for 10 min at 1500 rpm, filtered through low protein binding 0.45 μm syringe filter (Millipore, Bedford, MA) and partially purified through 25% wt/vol sucrose in Hepes-NaCl buffer by centrifugation at 36000 × *g *at 4°C for 2.5 hr. The pellet was resuspended overnight at 4°C in 10% sucrose in Hepes-NaCl buffer and used immediately or stored at -80°C.

### Incorporation of henipavirus envelope glycoproteins in the pseudotyped lentivirus particles

To measure the incorporation of the henipavirus F and G glycoproteins into pseudotyped HIV-1 particles, sucrose cushion purified particles were lysed in buffer containing 100 mM Tris-HCl (pH 8.0), 100 mM NaCl, 2% Triton X-100 and protease inhibitors at 4°C for 30 min. Samples were boiled in SDS-PAGE sample buffer with 2-mercaptoethanol and separated on a 4-20% Tris-Glycine gradient gels (Invitrogen), transferred to nitrocellulose, and probed with a cross-reactive polyclonal mouse antiserum to HeV G at a concentration of 1:25,000 or a rabbit polyclonal F_1 _specific antiserum at a concentration of 1:25,000.

### Pseudotyped virus infection assays

Receptor positive cell lines, seeded into 48-well plates at a concentration of 10^5 ^cells per well, were infected (transduced) with pseudovirus, normalized for p24 antigen content using the HIV-1 p24 EIA Kit from Beckman-Coulter, and all infection experiments were carried out in triplicate wells. No DEAE-Dextran or polybrene was used to facilitate fusion/infection by the pseudovirions. After infecting for 2.5-3 hr, the cells were washed and incubated for additional 48-72 hr. For luciferase encoding particles, cells were lysed with 0.5% Triton X-100 in PBS and a 50 μl aliquot of the lysate was assayed for luciferase activity using luciferase substrate (Promega, Madison, WI) on a Mikrowin luminometer (Berthold Technologies Model: Centro LB 960). For the GFP-encoding particles, the efficiency of infection was evaluated by counting the number of green cells 48 h post-infection using Olympus IX81 fluorescent microscope.

For inhibition of pseudotyped virus infection assays, pNL4-3-Luc-E-R^+ ^based virus particles pseudotyped with full-length NiV or HeV F and G envelope glycoproteins were pre-incubated with 2 μg each of NiVsG, mAb102.4 IgG, NiV-FC2 (fusion inhibiting) or Sc-NiV-FC2 (scrambled control peptide), recombinant soluble murine ephrin-B2 (rmEFN-B2/Fc) or recombinant soluble human ephrin-B3 (rhEFN-B3/Fc) at 4°C for 1 hr. Receptor positive 293T cells, seeded into 48-well plates (10^5 ^cells per well) were then infected with the various pre-treated pseudotyped virus particles and processed as above. All infection experiments were performed in triplicate.

## Competing interests

The authors declare that they have no competing interests.

## Authors' contributions

DK contributed to the development of the *Henipavirus *pseudovirus assay, designed and constructed all the expression constructs and carried out the experiments, identified the alternative splicing process in the original Hendra virus G encoding pCAGGs plasmid clone, interpreted data, and wrote the first drafts of the figures and manuscript. CCB conceived of and contributed to the development of the *Henipavirus *pseudovirus assay provided overall supervision and financial support and wrote and prepared the final versions of the figures and manuscript. Both authors read and approved the final manuscript.

## Supplementary Material

Additional file 1 **Splice site prediction in the pre-mRNA derived from the HeV G gene as cloned in pCAGGs**. EMBL-EBI Splice site prediction software http://www.ebi.ac.uk/asd-srv/wb.cgi?method=7 was used to check for the presence of splice donor sites in the G glycoprotein constructs cloned in the pCAGGs vector. Splice acceptor site present in the intron at position 1670-71 is shown on the left for all the constructs. On the right is shown the predicted splice donor site. Click here for file
