# Supplementary figures and images for: A Functional Henipavirus Envelope Glycoprotein Pseudotyped Lentivirus Assay System
Source: Virol J. 2010 Nov 12;7:312. doi: 10.1186/1743-422X-7-312 (PMC2994542; doi:10.1186/1743-422X-7-312)

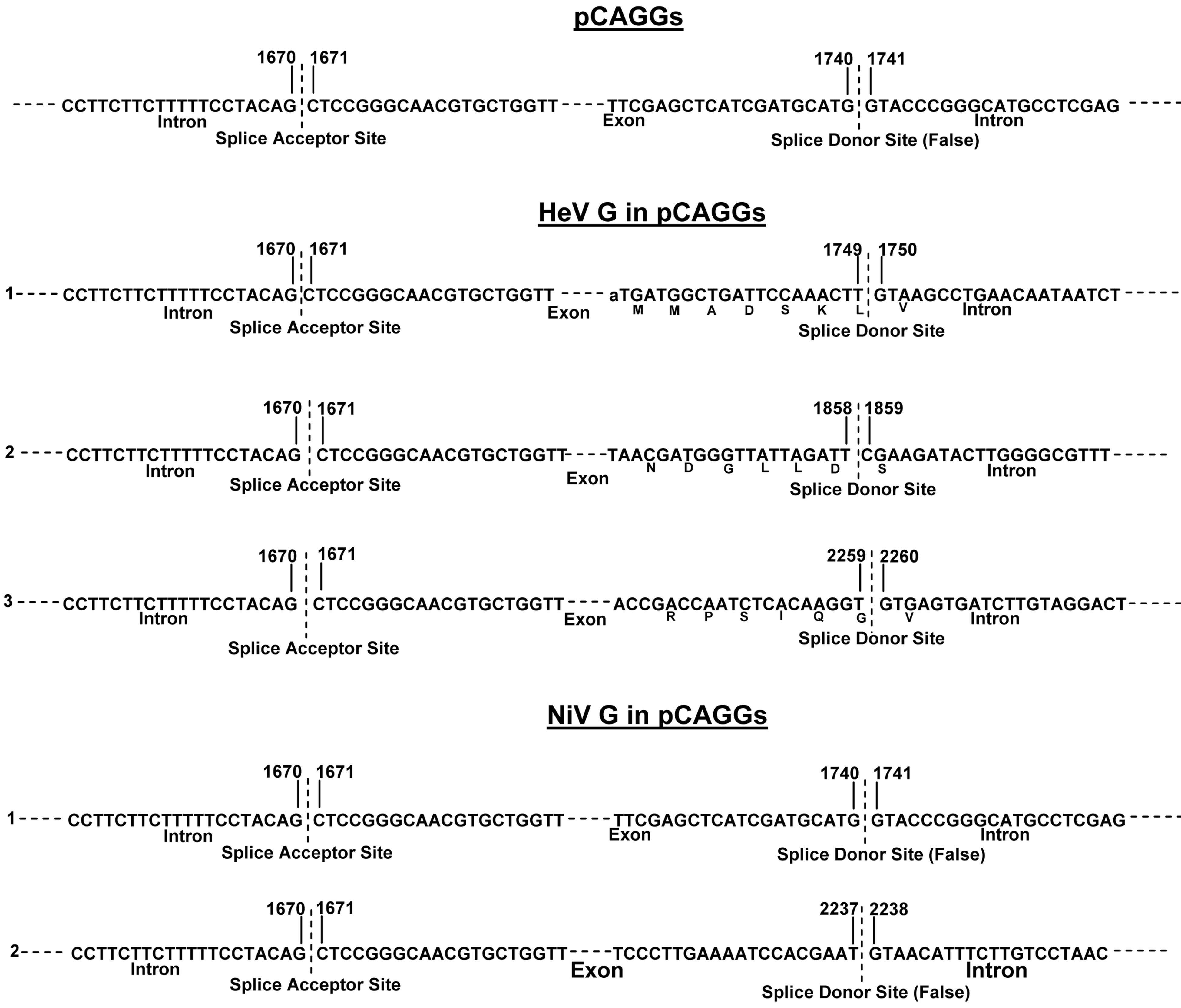

Supplement: Additional file 1 — Splice site prediction in the pre-mRNA derived from the HeV G gene as cloned in pCAGGs. EMBL-EBI Splice site prediction software http://www.ebi.ac.uk/asd-srv/wb.cgi?method=7 was used to check for the presence of splice donor sites in the G glycoprotein constructs cloned in the pCAGGs vector. Splice acceptor site present in the intron at position 1670-71 is shown on the left for all the constructs. On the right is shown the predicted splice donor site. [file 1743-422X-7-312-S1.TIFF]
